# Effect of Flavonoid Sources on Nutrient Intake and Digestibility, Rumen Fermentation, Growth Performance, Milk Production and Endoparasites in Ruminant Production: A Review

**DOI:** 10.1155/tswj/9915012

**Published:** 2026-03-31

**Authors:** Lindokuhle Christopher Mhlongo, Onke Hawu, Lwando Mbambalala, Lwazi Mwanda, Thando Tenza

**Affiliations:** ^1^ Department of Animal Science, University of the Free State, Bloemfontein, 9300, South Africa, ufs.ac.za; ^2^ Food Security and Saftey Niche Area, Faculty of Natural and Agricultural Sciences, School of Agricultural Sciences, North-West University, Mmabatho, 2735, South Africa, nwu.ac.za; ^3^ School of Interdisciplinary Research and Graduate Studies, College of Graduate Studies, University of South Africa, Pretoria, 0003, South Africa, unisa.ac.za; ^4^ Department of Animal and Wildlife Sciences, University of Pretoria, Pretoria, 0028, South Africa, up.ac.za; ^5^ Equitable Education and Economies Division, Human Sciences Research Council, Durban, South Africa, hsrc.ac.za

**Keywords:** flavonoid extracts, flavonoid-rich feeds, ruminant–flavonoid interaction

## Abstract

The objective of this review was the assessment of the effect of flavonoid sources on nutrient intake and digestibility, rumen fermentation, growth performance, milk production and endoparasites in ruminant production. The increasing human population is creating a greater demand for animal products to meet the protein needs. However, an increase in animal production is likely to force the overuse of synthetic antibiotics, which may intensify drug‐resistant pathogens. As a solution, there is a need to discover natural feed additives to improve environmental harmlessness, health and performance in ruminant production. This is because ruminants produce methane and suffer from infections such as gastrointestinal nematodes and mastitis that limit animal performance. Hence, there is a need to identify feed additives that decrease this gas without limiting nutrient utilization and animal performance. Flavonoid dietary inclusion is a potential solution to this challenge in ruminant production and has received research attention. However, there is a preliminary understanding of the effect of inclusion on effectiveness in decreasing methane production and pathogens, improving rumen fermentation, milk production and growth performance in ruminants. Therefore, there is a need for the identification of the effect of flavonoid inclusion on the abovementioned parameters for the detection of research limitations requiring assessment for the identification of a single flavonoid inclusion with a holistic effect on the production parameters of ruminants.

## 1. Introduction

The world population growth rate is increasing at a higher rate than that of animal production. The consequence of the mismatch in population growth and animal production rates is pressuring the animal production industry to intensify which mayincrease methane production. Furthermore, intensification of animal production would increase the use of synthetic chemicals to control animal pathogens, stimulate growth and control methane emissions. Intensified synthetic drug use has the potential to increase the number of resistant microbes. Drug‐resistant microbes would lower animal performance and lead to an inability to meet animal protein requirements of the increasing world population [1]. In addition, intensification of animal production could increase feed costs due to the use of human edible feed in animal production [2].

Therefore, there is a need to find alternative ways to control the expected increase in resistance as multi–drug‐resistant pathogens such as *Escherichia coli* have been noted due to overuse of commercial antibiotics [3]. Flavonoid sources are an alternative to conventional drugs for controlling pathogens, limiting animal production, such as *E. coli* [4]. This phytochemical has varying modes of action compared to those of commercial feed additives. Thus, this phytochemical can control pathogens affecting livestock production without resistance development [5]. For instance, communal farmers use flavonoid‐rich plants to control pathogens that lower animal performance [6]. Another advantage of flavonoid‐rich plants such as *Leucaena leucocephala* [7] or *Moringa oleifera* [8] is their potential to serve as conventional feed replacements in animal production.

Research has been conducted over the years on the impact of flavonoid plants on animal performance parameters. However, there is a lack of a summary of dietary inclusion ranges of flavonoids that can be used to enhance growth performance, production performance, health parameters and fermentation parameters in ruminants. Future research needs to identify flavonoid inclusion ranges that improve different parameters of animal production, nutrition and health on different pathogens and growth parameters in animal production. This could lead to the identification of a dietary inclusion range that optimizes the majority of animal performance parameters in ruminants. This intervention will aid in the identification of a single dose range that can control and promote production parameters holistically, which can be identified through the understanding of the effect of flavonoids on all production parameters concerned. As a result, the objective of this review was to assess the effect of dietary inclusions of flavonoid sources on growth performance, rumen fermentation, endoparasite and milk production in ruminants.

## 2. Effect of Flavonoid Sources on Nutrient Intake

Flavonoid sources have various effects on nutrient intakes as they either increase, decrease or do not affect nutrient intakes (Table 1). Intakes of dry matter (DM) increased by inclusion of sea‐buckthorn flavonoid [12], red propolis extract [13] or licorice root extract [14]. The increase in nutrient intake associated with flavonoid extract may be because they possess no extra feed components such as fibre that may depress nutrient intake. Therefore, the absence of feed components may increase feed eating times since fibre decreases feed eating time. Some flavonoid extracts such as powder may possess lower fibre content, which may even lead to no effect of flavonoid on nutrient intake. Another past result showed that mangosteen peel powder had no effect on nutrient intake [10].

**TABLE 1:** Effect of flavonoid inclusions on nutrient intake.

Source	Animal	Inclusion	Response	Reference
Mulberry leaves	Sheep	0 or 2 g/d	• DM and OM intakes were not affected	[9]
Mangosteen peel powder	Goats	0, 4.125%	• DM and OM intakes were not affected	[10]
Wolfberry branches and leaves	Sheep	0, 3, 6, 9 or 12%	• Feed intake increased by 6 and 9%, but the 12% inclusion decreased feed intake	[11]
Sea‐buckthorn flavonoid extract	Lambs	0, 0.1, 0.3 or 0.5%	• DM intake increased	[12]
Red propolis extract	Sheep	0 or 3 g/d	• Feed intake increased	[13]
Licorice root extract	Sheep	0 or 4.5%	• DM intake increased	[14]

This also explains why flavonoid sources in feed form tend to decrease feed intake until a certain inclusion, whereby feed intake decreases. For instance, wolfberry leaves and branches increased intake between 3% and 9% inclusion but decreased intakes at 12% inclusions [11]. This result may suggest that the fibre components of wolfberry leaves and branches were increased to a level of decreasing feed intake at 12%. Therefore, it suggests that inclusions of flavonoid‐rich feeds may sometimes not affect if the inclusion is less than 12%, lower fibre content. This may explain why DM and organic matter (OM) intakes remained unaffected when mulberry leaves were included [9]. The influence of flavonoid sources on the feeding behaviour may also explain the absence of flavonoid impact on feeding behaviour. It has been reported previously that flavonoid source inclusion does not affect eating time [15].

## 3. Effect of Flavonoid Sources on Nutrient Digestibility

Flavonoid sources tend to increase the digestibility of nutrients (Table 2). However, the influence of flavonoid sources on nutrient digestibility is specific in terms of the flavonoid source type and nutrient. Feed is represented on a DM basis. It was found that sea‐buckthorn flavonoid extract increases DM digestibility [12], whereas it was reported that mangosteen peel powder increased only OM digestibility [10]. DM and OM are feed components that encompass nutrients such as crude protein (CP), ether extract (EE), neutral detergent fibre (NDF) and acid detergent fibre (ADF). However, it is limiting to rely on DM digestibility or OM as a representative nutrient for the digestibility of other nutrients such as CP, ADF, NDF and EE, which have unique responses to flavonoid sources.

**TABLE 2:** Effect of flavonoid sources on the nutrient digestibility.

Source	Animal	Inclusion	Response	Reference
Mulberry leaves	Calves	0 or 4 g/d	• ADF, NDF and EE digestibility increased	[16]
			• Digestibility of DM, CP and OM was not affected	

Mangosteen peel powder	Goats	0 or 4.125%	• Increased OM digestibility only	[10]

Mulberry flavonoids	Sheep	0 or 2 g/d	• Digestibility of DM and OM was not affected	[9]

Wolfberry branches and leaves	Sheep	0, 3, 6, 9 or 12%	• CP digestibility was increased compared to the control, but the 3% and 6% inclusions had the highest CP digestibility	[11]

Napier grass	Goats	0 or 50%	• Digestibility of DM, OM, EE, CP, NDF and ADF was higher in fresh than in silage Napier grass	[17]

Sea‐buckthorn flavonoid extract	Lambs	0, 0.1, 0.3 or 0.5%	• DM digestibility increased with sea‐buckthorn supplementation and was the highest at 0.3% inclusion	[12]

For instance, mulberry leaves were observed for increasing only the digestibility of ADF, NDF and EE in calves [16]. Moreover, Wolfberry branch leaves have previously been observed for increasing the digestibility of CP [11]. On the other hand, it was demonstrated that mulberry flavonoids increase only DM and OM digestibility in sheep [10]. This suggests that there is an animal species and flavonoid source interaction. For instance, sheep have demonstrated lower digestibility of DM, OM, CP, EE and NDF than goats [18]. Therefore, it is possible that flavonoids may increase the digestibility of most nutrients only when added to diets fed to animal species such as goats, which are associated with higher nutrient digestibility. This is supported by past findings which noted that Napier grass increased the digestibility of DM, OM, EE, CP, NDF and ADF in goats [19]. In addition, the rumen has low functionality, and the abomasum has a younger age [20]. Therefore, it is possible for nutrient digestibility to be affected by animal age. Calves, lambs or goat kids are more likely to fail to express an increase in nutrient digestibility due to an underdeveloped digestive system.

## 4. Effect of Flavonoid Sources on Rumen Methane Production

Inclusions of flavonoid sources reduce methane production (Table 3). Flavonoid sources depress methane production regardless of the flavonoid type and animal species. Methane depression demonstrates a dose‐dependent response. The explanation for the dose‐dependent effect may be the increase in flavonoid content with inclusion. The substrate involved in the supplementation of flavonoids on methane production plays a role. For instance, it was demonstrated that quercetin inclusion levels have higher methane production under a concentrate substrate compared to a straw substrate [26].

**TABLE 3:** Effect of flavonoid sources on methane production in ruminants.

Source	Animal	Inclusion	Response	Reference
*Cassia fistula* leaf powder	Goats	0, 15, 30 or 60 mg/g DM	• Methane gas production *in vitro* decreased with increasing inclusion of *Cassia fistula*	[21]

*Piper betle* powder	Goat	0, 15, 30, 45 and 60 mg DM	• Methane gas production decreased with increasing *Piper betle* powder	[22]

Green tea ethanol extract	Cows	0, 250, 500, 750 or 1000 μg/mL	• Methane gas production decreased with increasing green tea extract inclusions	[23]

Mulberry leaf flavonoids	Sheep	0 or 2 g/d	• Methane decreased with increasing flavonoid inclusion	[24]

*Lycium barbarum* seed	Sheep	0, 2.5 or 7.5%	• Methane production decreased with the inclusion of *L. barbarum* seed, residue or twigs	[25]

Quercetin or rutin	Cows	0, 50 or 100 μmol/L	• The methane production decreased with the increase in inclusion of quercetin or rutin	[26]
			• Methane production decreased more if the substrate was straw or hay than concentrate	

Rumen microbes are pH‐sensitive and can only survive when the pH level is neutral. It has been reported that rumen pH has a positive relationship with flavonoid concentration level [27]. It has also been noted that flavonoid source increases the population of protozoa while decreasing bacteria [28]. This suggests that protozoa populations feed on rumen bacteria. Therefore, it is possible that the depression of methane is due to protozoal populations, which reduce their associated methanogens, resulting in decreased methane production. Future studies should ascertain whether there is an animal species effect on the response of methane production to flavonoid source inclusions. Moreover, there is a need to assess this research focus *in vivo*, as most of the current findings are based on the *in vitro* findings.

## 5. Effect of Flavonoid Sources on Rumen Volatile Fatty Acids

The effects of flavonoid inclusion on volatile fatty acid responses appear to be inconsistent. This is because some flavonoid sources increase or decrease only acetate, propionate or total volatile fatty acids (Table 4). Also, different types of flavonoid extract produce conflicting results on volatile fatty acid response. Total volatile fatty acids and acetic acids have been previously increased with the inclusion of mangosteen peel [10], sea‐buckthorn flavonoids [12] or mulberry flavonoids [29]. On the other hand, it has been shown that flavonoid sources only increase total volatile fatty acids. For example, it has been demonstrated that mulberry leaf flavonoids only increased total flavonoids [24]. The increase in total volatile fatty acids or acetate may be due to the fact that nitrogen ammonia (ammonia‐N) remained unaffected by the inclusion levels used. It has been reported that an increase in ammonia‐N correlated with an increase in total volatile fatty acids and acetate production in dietary inclusions of mulberry flavonoids [29].

**TABLE 4:** Effect of flavonoid sources on rumen volatile fatty acids.

Source	Animal	Inclusion	Response	Reference
Mangosteen peel	Goat	0 or 4.125%	• Total volatile fatty acids (VFAs) and acetic acids increased	[10]

Sea‐buckthorn flavonoids	Sheep	0, 0.1, 0.3 or 0.5%	• Total VFAs and the acetate to propionate ratio increased	[12]

Mulberry flavonoids	Buffaloes	0, 15, 30 or 45 g/d DM	• Acetate and total volatile fatty acids increased with increasing mulberry flavonoids	[29]
			• Propionate increased only at 30 and 45 g/d DM	

Mulberry leaf flavonoids	Sheep	0 or 2 g/d	• Total VFAs increased	[24]

Quercetin or rutin	Cows	0, 50 or 100 μmol/L	• The total VFAs were not affected.	[26]

Quercetin	Cattle	0, 0.5, 1 or 1.5%	• None of the VFAs were affected	[30]

Hesperidin, isonaringin, naringin, neoeriocitrin, neohesperidin or poncirin	Steers	0 or 0.02% DM	• Propionate increased while acetate decreased	[31]

Ammonia‐N serves as a growth factor for rumen microbes, which can result in higher production of volatile fatty acids. Also, the gas production rate associated with the digestion of the fibre component of the feed play a role in how volatile fatty acids respond to flavonoid inclusions. It has been reported that quercetin inclusion increases the rate of gas production of the fibrous component [30]. Acetate is produced from fibre digestion. Therefore, it is possible that the lack of the effect of flavonoids on the rate of gas production associated with fibre digestion results in no response to volatile fatty acids. For instance, quercetin or rutin inclusions did not affect the response of total volatile fatty acids [26]. Quercetin inclusions at a different inclusion also did not affect volatile fatty acids [30]. Therefore, flavonoids may not be adequate to elicit a reaction, which could explain why flavonoid sources have little effect on volatile fatty acids. However, it has been demonstrated that propionate may increase at the expense of acetate. There was an observation of inclusions of hesperidin, isonaringin, naringin, neoeriocitrin, neohesperidin or poncirin increasing propionate while decreasing acetate production [31]. This may be due to the substrate being more concentrated than roughage [26].

## 6. Effect of Flavonoid Sources on Rumen Microbial Populations

Different types of flavonoids tend to mainly decrease rumen microbial populations (Table 5). The depression of rumen microbes by flavonoid sources may lead to rumen microbes, which are important fibre digestibility. Therefore, the identification of an optimal flavonoid source that does not affect microbial populations is important for normal rumen function. However, some flavonoid sources such as lucerne flavonoid extract [32] and citrus flavonoid extract [34] did not affect rumen microbes. The differences in whether flavonoid source decreases rumen microbes or not may rely on the dose.

**TABLE 5:** Effect of flavonoid sources on rumen microbial populations.

Source	Animal	Inclusion	Response	Reference
Lucerne flavonoid extract	Cows	0, 20, 60 or 100 mg/kg BW	• Rumen microbes were not affected (*S. bovis*, *R. flavefaciens* or *B. fibrisolvens*)	[32]
Propolis extract	Cow	0, 25, 50 or 75%	• Rumen microbes decreased	[33]
Citrus flavonoid peel extract	Cows	0 or 2%	• Rumen microbes were not affected except for methanogens which decreased	[34]
Quercetin or catechin	Cows	0 or 4.5%	• Rumen microbes decreased, but catechin had a higher rate of microbe depression than quercetin	[35]

It seems that inclusions of more than 2% are detrimental to the rumen microbial populations (Table 5). The type of flavonoid source affects the response of rumen microbes. For example, catechin had a higher rate of microbial depression than quercetin [35]. Quercetin and catechin are fractions of flavonoids, which are found in flavonoid sources. However, it is possible that the observation of the depression of rumen microbes may be due to the use of a flavonoid source that has more catechin than quercetin. Therefore, it might be beneficial to investigate the ratios of different flavonoid types on rumen microbes for the identification of an optimal ratio that is less detrimental.

There is also a need to assess other microbial species’ response to flavonoid inclusions. This is because studies assessing the influence of flavonoid sources on microbial populations do not assess the response of methanogens. Hence, it is important to assess the influence of flavonoids on methanogens to find a way to decrease methane production. This is as it was noted that citrus flavonoid peel extracts decreased methanogens [34]. Thus, flavonoids seem to indirectly contribute to the depression of methane production, and the investigation into their effects on methanogens needs expansion.

## 7. Effect of Flavonoid Sources on Growth Performance in Young Ruminants

Various flavonoid sources increase growth parameters such as average daily gain (ADG), final bodyweight (final BW) and average daily feed intake in small ruminants (Table 6). However, the results show that flavonoid sources such as flavonoid extract may not be effective in enhancing growth performance parameters in calves [37]. The dose of flavonoid sources seems to be influencing the efficacy of flavonoid sources. Polyherbal flavonoid additive increased ADG at an inclusion range of 0.1–0.2% but decreased ADG at an inclusion range of 0.3% [36]. This suggests that the polyherbal flavonoid additive may contain flavonoids that are too high. Conversely, inclusion of sea‐buckthorn flavonoids at an inclusion range of 0.1–0.5% improved BW and ADG [12].

**TABLE 6:** The effect of flavonoid sources on growth performance.

Source	Animal	Inclusion	Response	Reference
Polyherbal flavonoid additive	Lambs	0, 0.1, 0.2 or 0.3%	• ADG increased with the inclusion of 0.1 and 0.2%, while 0.3% decreased it	[36]
Flavonoids	Calves	0, 7.3 × 10^−4^, 7.3 × 10^−3^ or 7.3 × 10^−5^ g/kg BW	• None of the growth performance variables were affected	[37]
Sea‐buckthorn	Lambs	0, 0.1, 0.3 or 0.5%	• Final BW increased while ADG decreased	[12]
Bioflavex	Lambs	0, 0.04 or 0.08%	• Feed conversion ratio decreased, growth rate increased, and weight gain increased	[38]
Ampelopsis grossedentata flavonoids	Goat kids	0 or 0.1%	• ADFI and final BW increased	[39]

This further confirms that sea buckthorn at a higher dose than that of polyherbal flavonoid enhances growth performance due to their flavonoid content potentially being lesser compared to that of polyherbal flavonoid. Thus, it is important to identify flavonoid sources that are effective at minimal inclusion rates to prevent depression of growth parameters. Flavonoid sources such as bioflavex are potential flavonoid sources that could enhance growth performance at low inclusion levels as they have been noted for improving feed conversion ratio, growth rate and ADG at inclusion levels lower that 0.1% [38].

The reason for the increase in BW parameters and growth rate may be because at 0.1% or lower flavonoid concentrations maximize nutrient intake, which is needed for the enhancement of BW and growth rate. For instance, *Ampelopsis grossedentata* flavonoids increased feed intake and final BW at an inclusion rate of 0.1% [39]. The increase in feed intake may be due to the ability of certain flavonoid sources to enhance rumen development. It is possible that the influence of flavonoids on growth performance parameters in young ruminants may require different inclusions in pre‐ and postweaning periods. This is because it has been demonstrated that mulberry flavonoids did not affect preweaning ADG but increased postweaning ADG [41]. Therefore, there is a need for the identification of the effect of flavonoid sources on the post‐ and preweaning growth stages.

## 8. Effect of Flavonoid Sources on Milk Parameters

Flavonoid sources increase milk production and composition with milk parameter‐specific effect (Table 7). The flavonoid dietary inclusion of 6% chaya pellets increased percentages of protein and fat and milk yield [41]. Conversely, the same inclusion of 10% of *Broussonetia papyrifera* only increased milk yield [43]. This suggests that milk components have varying responses to flavonoid sources. The differences may be caused by the fact that different flavonoid sources have different flavonoid fractions, which then can lead to a nonhomogeneous response of milk parameters. Furthermore, the effect of different flavonoid sources depends on the dose. Thus, it is expected that doses of less than 1% of flavonoid sources will lack an effect, while some present an effect on milk parameters. For instance, 0.004% inclusion of *Scutellariae radix* flavonoid extract results in no response in milk parameters [42], while inclusion range of 0.2–0.7% of citrus flavonoid extract increased milk yield [45].

**TABLE 7:** Effect of flavonoid sources on milk production and composition.

Flavonoid sources	Animal	Inclusion	Response	Reference
Chaya pellets	Dairy cows	0 or 6%	• Milk yield and percentage of fat and protein increased	[41]
Scutellariae radix flavonoid extract	Dairy cows	0 or 0.004%	• Milk production or composition was not affected	[42]
*Broussonetia papyrifera*	Dairy goats	0 or 10%	• Milk composition was not affected, but milk yield increased	[43]
Hesperidin or naringin	Dairy goats	0 or 0.6%	• Milk composition or milk yield was not affected	[44]
Citrus flavonoid extract	Dairy cows	0, 0.2, 0.5 or 0.7%	• Only milk yield increased	[45]
Moringa leaf flavonoids	Dairy cows	0, 50 or 100 mg/BW	• Milk protein increased	[46]

There is a need to identify a flavonoid source with a uniform impact of milk parameters. Thus, there is a need to combine flavonoid sources that are specific for milk composition with those that are specific for milk yield. Future studies should identify the optimal ratio of the above‐mentioned types of flavonoids that improve all milk parameters. Since results show that milk composition is less likely to increase from flavonoid source inclusions, there is a need to dose per BW. For instance, past results also demonstrate that the supplementation of flavonoid sources per BW of animals improves milk protein content [46].

## 9. Effect of Flavonoid Sources on Mastitis Infection

Mastitis infections decrease with increasing inclusion of flavonoid sources (Table 8). Different types of flavonoid sources can reduce mastitis infections. The effective inclusions of flavonoid sources are 120 mg/d [47] and 10–100 μg/mL [48]. It is also demonstrated that postdipping with flavonoid sources such as green propolis and aloe vera decreases mastitis. The efficacy of flavonoid sources was found to be dependent on the duration of their application. For instance, it has been demonstrated that the efficacy of *Scutellaria baicalensis* flavonoids against somatic cell count increases 60 days after postapplication [49]. This suggests that there is a need identify the optimal duration of application suitable for flavonoid sources that are used to control mastitis. Therefore, the antimastitis efficacy of flavonoid sources may be increased by the identification of the optimal duration of their application.

**TABLE 8:** Effect of flavonoid sources on the mastitis infections.

Flavonoid sources	Animal	Inclusion	Response	Reference
Baicalin flavonoid	Dairy cows	0 or 120 mg/d	• Somatic cell count decreased	[47]
Quercetin	Dairy cows	10, 50 or 100 μg/mL	• Inflammation of the mammary gland infected with *S. aureus* decreased	[48]
*Scutellaria baicalensis* flavonoids	Dairy cows	0, 5 or 60 d of treatment	• Somatic cell count decreased in the 60‐d treatment	[49]
Green propolis and aloe vera	Dairy cows	Postdipping or no postdipping with treatment	• Mastitis was decreased by postdipping with the treatment	[50]

## 10. Effect of Flavonoid Sources on Gastrointestinal Nematodes

Different types of flavonoid sources reduce gastrointestinal nematodes (Table 9). There are different flavonoid sources such as flavonoid extract, flavonoid fractions or flavonoid‐rich leaves are used to control gastrointestinal nematodes. The efficacy range of flavonoid sources seems to range from 0.62% to 20% (Table 9). There seems to be a dose‐dependent response mainly within this flavonoid inclusion range. The efficacy may be due to the increase in flavonoid amount per increase in inclusion level of the flavonoid [53]. Most of the findings on the effect of flavonoids are based on *in vitro* studies (Table 9). It has been noted that *in vitro* efficacy of flavonoids as anthelmintics is lower *in vivo*. Hence, it is important to identify an extractant that is associated with higher anthelmintic efficacy of flavonoid sources.

**TABLE 9:** Effect of flavonoid sources on the gastrointestinal nematodes.

Flavonoid source	Animal	Inclusion	Response	Reference
Ivermectin, quercetin or quercetin + ivermectin	Sheep	5, 7 or 9 d post‐treatment	• Quercetin improves the anthelmintic efficacy of ivermectin	[51]
			• Quercetin alone decreased faecal egg count (FEC)	

*Lysiloma acapulcensis* as aqueous extract or fraction; and ethyl acetate fraction	Sheep	6.2, 12.5, 25 or 50 mg/mL	• The inclusions of 6.2–25 mg/mL decreased FEC	[52]

*Psidium guajava* hydroalcoholic extract	Sheep	0.062, 0.125, 0.25 or 5%	• Inhibition of FEC increased with treatment inclusion increment	[53]

*Moringa oleifera* leaves	Sheep	0.5, 1, 5, 10 or 20%	• FEC inhibition increased with increment of treatment inclusion	[54]

*Acacia farnesiana* flavonoids	Sheep	0.625, 2.5 or 5%	• FEC decreased with an increase in inclusions, and aqueous extract had lower efficacy than hydroalcoholic extract	[55]

It has been shown that hydroalcoholic extract has higher efficacy than aqueous extracts [55]. This would assist in developing flavonoid sources as boosters of conventional anthelmintics such as ivermectin, which have a decreasing efficacy due to resistant gastrointestinal nematodes. For instance, it has been demonstrated that quercetin improves the efficacy of ivermectin [51]. Moreover, most studies focus on assessing the efficacy of flavonoid sources on gastrointestinal nematodes in sheep. Therefore, there is a need to expand research on the effect of flavonoid sources on goat gastrointestinal nematodes as well. This is because flavonoid sources have lower efficacy in goats compared to sheep gastrointestinal nematodes [56].

Some of the studies assessed the response of mixed gastrointestinal nematodes [52, 53]. Such an approach limits the understanding of the effect of flavonoid sources on other gastrointestinal nematode species. However, other studies have assessed response to flavonoid source per specific gastrointestinal nematode species. For instance, some studies demonstrated the assessment of flavonoid extract on *Haemonchus contortus* only [51, 54, 55]. Species‐specific differences among gastrointestinal nematodes are important for understanding the influence of flavonoids on individual species. It has been noted that different gastrointestinal nematode species respond differently to flavonoid sources.

## 11. Conclusions

This review demonstrated that flavonoid sources exert conflicting results on nutrient intake. However, it also showed that flavonoid sources increase nutrient digestibility with a nutrient‐specific effect. Moreover, flavonoid sources decrease methane production, rumen microbes, mastitis infections and gastrointestinal nematode infection. Furthermore, flavonoid sources increase milk parameters, growth performance and rumen volatile fatty acids with an effect that is unique for each variable. However, there is a need to investigate the dose and type effects of flavonoids on performance parameters under various parameters that influence the response of ruminant parameters. Some of those variables include substrate type, forage to concentrate ratio, ruminant species and animal age.

## Funding

No funding was received for this study.

## Conflicts of Interest

The authors declare no conflicts of interest.
